# Nrf2 deficiency decreases NADPH from impaired IDH shuttle and pentose phosphate pathway in retinal pigmented epithelial cells to magnify oxidative stress‐induced mitochondrial dysfunction

**DOI:** 10.1111/acel.13444

**Published:** 2021-07-27

**Authors:** Marisol Cano, Sayantan Datta, Lei Wang, Tongyun Liu, Miguel Flores‐Bellver, Mira Sachdeva, Debasish Sinha, James T. Handa

**Affiliations:** ^1^ Wilmer Eye Institute Johns Hopkins School of Medicine Baltimore MD USA; ^2^ Department of Ophthalmology University of Pittsburgh School of Medicine Baltimore MD USA

**Keywords:** aging, mitochondria, oxidative stress, reactive oxygen species

## Abstract

The nuclear factor‐erythroid 2‐related factor‐2 (Nrf2), a major antioxidant transcription factor, is decreased in several age‐related diseases including age‐related macular degeneration (AMD), the most common cause of blindness among the elderly in western society. Since Nrf2’s mito‐protective response is understudied, we investigated its antioxidant response on mitochondria. Control and Nrf2‐deficient retinal pigmented epithelial (RPE) cells were compared after treating with cigarette smoke extract (CSE). Mitochondrial antioxidant abundance and reactive oxygen species (ROS) were quantified. Mitochondrial function was assessed by TMRM assay, NADPH, electron transport chain activity, and Seahorse. Results were corroborated in Nrf2^−/−^ mice and relevance to AMD was provided by immunohistochemistry of human globes. CSE induced mitochondrial ROS to impair mitochondrial function. H_2_O_2_ increase in particular, was magnified by Nrf2 deficiency, and corresponded with exaggerated mitochondrial dysfunction. While Nrf2 did not affect mitochondrial antioxidant abundance, oxidized PRX3 was magnified by Nrf2 deficiency due to decreased NADPH from decreased expression of IDH2 and pentose phosphate pathway (PPP) genes. With severe CSE stress, intrinsic apoptosis was activated to increase cell death. PPP component TALDO1 immunolabeling was decreased in dysmorphic RPE of human AMD globes. Despite limited regulation of mitochondrial antioxidant expression, Nrf2 influences PPP and IDH shuttle activity that indirectly supplies NADPH for the TRX2 system. These results provide insight into how Nrf2 deficiency impacts the mitochondrial antioxidant response, and its role in AMD pathobiology.

## Graphical abstract

The antioxidant transcription factor Nrf2 does not regulate mitochondrial antioxidant gene expression, but instead genes in the pentose phosphate pathway and the IDH shuttle, which together provide NADPH to the mitochondria for the TRX2 system to neutralize H_2_O_2_. With Nrf2 deficiency, which occurs in age‐related macular degeneration, mitochondria become damaged by inadequately neutralized H_2_O_2_ after smoking.

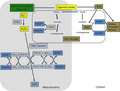

**Abbreviations**
ARE: antioxidant response element
AMD: Age‐related macular degeneration
CSE: Cigarette smoke extract
ECAR: Extracellular acidification rate
ETC: Electron transport chain
G6PD: Glucose‐6‐phosphate dehydrogenase
GAPDH: Glyceraldehyde‐3‐Phosphate Dehydrogenase
GCLM: Glutamate‐Cysteine Ligase Modifier Subunit
GLUD‐1: Glutamate Dehydrogenase 1
GSH: Glutathione
HIF1a: Hypoxia inducible factor‐1 alpha
IDH‐1: Isocitrate dehydrogenase‐1
IDH‐2: Isocitrate dehydrogenase‐2
iPS‐RPE: induced pluripotent stem cell‐RPE
IVT: intravitreal
ME‐3: malic enzyme‐3
MMP: mitochondrial membrane potential
NADH: Nicotinamide adenine dinucleotide
NADPH: Nicotinamide adenine dinucleotide phosphate
NNT: Nicotinamide nucleotide transhydrogenase
Nrf2: Nuclear‐factor‐erythroid 2‐related factor‐2
NQO‐1: NAD(P)H Quinone Dehydrogenase 1
OCR: oxygen consumption rate
O2−: Superoxide anion
ONOO−: Peroxynitrites
PGD: 6‐phosphogluoconate dehydrogenase
PPP: Pentose phospate pathway
PRX3: Peroxiredoxin‐3
ROS: Reactive oxygen species
RPE: Retinal pigment epithelium
SIRT3: Sirtuin‐3
SLC25A1: Solute Carrier Family 25 Member 1
SOD‐2: superoxide dismutase‐2
SRX: Sulfiredoxin
TALDO1: transaldolase‐1
TKT: Transketolase
TRX2: Thioredoxin‐2
VDAC: Voltage‐dependent anion channel

## 1 INTRODUCTION

While reactive oxygen species (ROS) at physiologic levels are essential for signaling, the added burden of ROS from cigarette smoking markedly augments oxidative stress. Aging impairs the antioxidant response to increase ROS. Chronic oxidative stress contributes to aging and age‐related disease due to cellular dysfunction (Harman, 1956). Age‐related macular degeneration (AMD), the leading cause of blindness among the elderly, a disease with oxidative stress as a prominent etiologic factor, is unfortunately without effective treatment for early disease (Age‐related Eye Disease Study Research Group, 2001).

The retinal pigment epithelium (RPE) is harmoniously located between the photoreceptors and choriocapillaris. The RPE interacts with light‐detecting photoreceptors to maintain their health and provide trophic support to the choriocapillaris. To meet these functions, the RPE have a well‐developed mitochondrial network. The RPE has a robust antioxidant system because it resides in a high oxidative stress environment due to the high oxygen tensions needed to match its robust metabolism, and its unique photo‐oxidative exposure with vision (Alder & Cringle, 1985; Rozanowska et al., 1995). The aging RPE becomes susceptible to oxidative stress, and progressive oxidative damage can induce RPE dysfunction (Beatty et al., 2000). When dysfunctional, the RPE is centrally involved in AMD pathobiology and can ultimately die by apoptosis (Del Priore et al., 2002; Dunaief et al., 2002). Cigarette smoking, a mixture of chemical oxidants, is the strongest environmental risk factor for AMD (Smith et al., 2001; Tomany et al., 2004). In evaluating the global transcriptional response by RPE cells to cigarette smoke, we found that the antioxidant response is critical for survival and that mitochondria are vulnerable to injury (Cano et al., 2014; Wang et al., 2020). Given that mitochondrial dysfunction and cigarette smoking are implicated in early AMD (Karunadharma et al., 2010; Lin et al., 2011; Terluk et al., 2015), understanding how mitochondria protect against oxidative stress could lead to new therapy.

The nuclear factor‐erythroid 2‐related factor‐2 (Nrf2) transcription factor is well known for regulating perhaps the most comprehensive antioxidant response in all cell types (Sykiotis & Bohmann, 2010). Besides regulating antioxidant genes, Nrf2 influences NADPH abundance that is used for reducing oxidized antioxidants by regulating malic enzyme‐1 (ME‐1), isocitrate dehydrogenase‐1 (IDH1), glucose‐6‐phosphate dehydrogenase (G6PD), and 6‐phosphogluoconate dehydrogenase (PGD) (Lee et al., 2003; Mitsuishi et al., 2012; Thimmulappa et al., 2002; Wu et al., 2011). Nrf2 affects mitochondrial bioenergetics as well, by regulating the availability of substrate for electron transport chain (ETC) 1 and 2 (Holmstrom et al., 2013). In addition, Nrf2 influences fatty acid oxidation and ATP synthesis (Holmstrom et al., 2013; Ludtmann et al., 2014). These factors collectively, affect mitochondrial health. Despite this understanding, the impact of Nrf2 on mitochondrial antioxidant protection is understudied. Our lab demonstrated that Nrf2 is decreased in the RPE with early AMD and smoking (Wang et al., 2014), as in other oxidative stress diseases like emphysema (Suh et al., 2004; Suzuki et al., 2008). How impaired Nrf2 signaling influences the RPE, especially after exposure to cigarette smoke, which preferentially damages mitochondria, is unclear. The purpose herein was to evaluate the mito‐protective response to smoke and impaired Nrf2 in the RPE.

## 2 RESULTS

### 2.1 Nrf2 deficiency impairs the cytoplasmic antioxidant response

**FIGURE 1:**
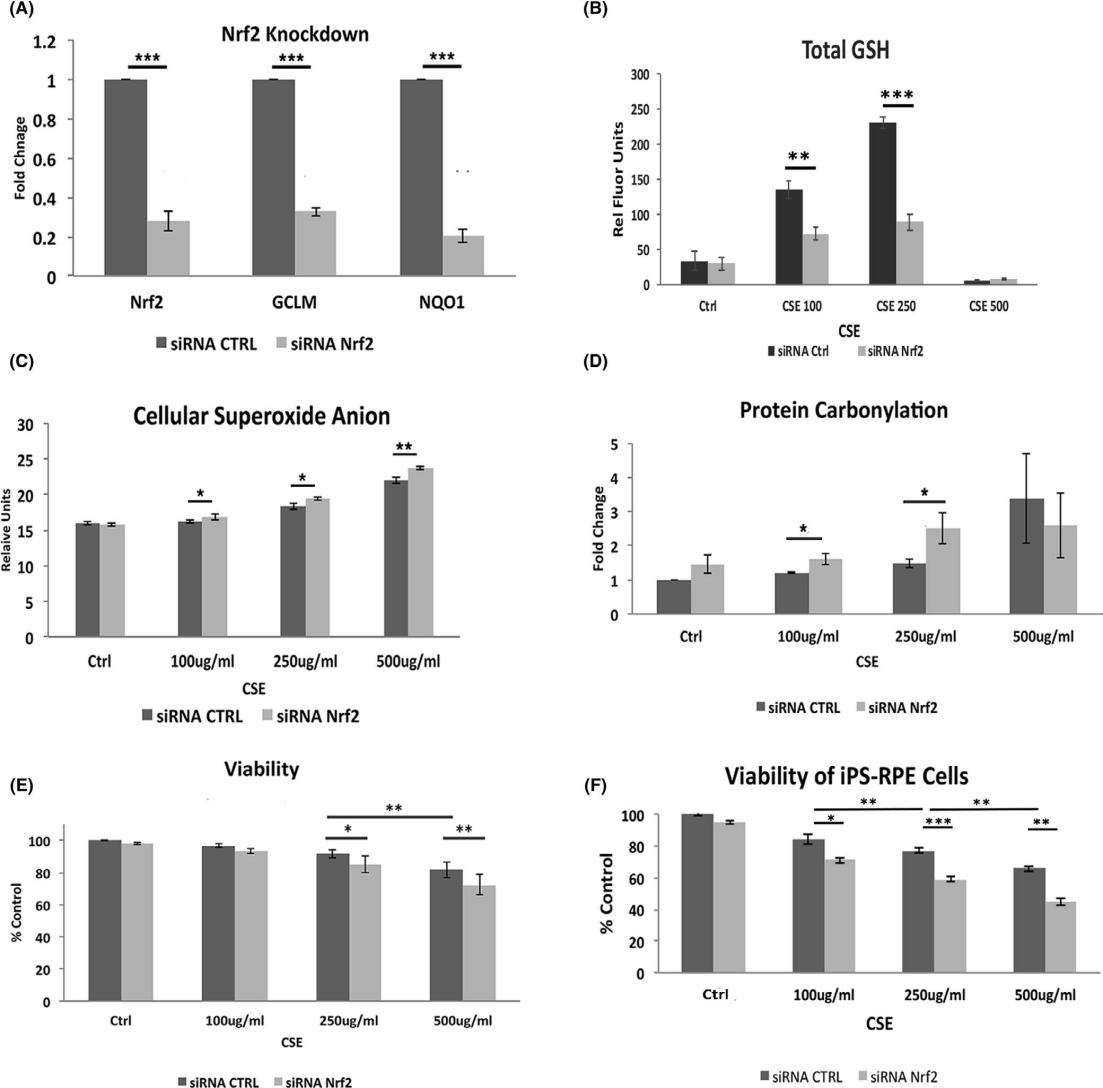
Nrf2 deficiency increases oxidative stress. A, Nrf2 knockdown (KD) by siRNA decreases Nrf2, GCLM, and NQO1 expression using RT‐qPCR. B, Dose dependent increase in total GSH with CSE is reduced with Nrf2 KD. C, ROS increases with CSE, and is magnified by Nrf2 KD using DHE assay. D, Protein carbonylation increases with CSE to a greater extent in cells with Nrf2 KD. E, Cell death by the live‐dead assay, manifests at 250 μg/ml CSE and increases with Nrf2 KD. F, Cell viability is also decreased with CSE and magnified with Nrf2 KD in iPS‐RPE cells. *p < 0.05, **p < 0.01, ***p < 0.001

RPE cells treated with an siRNA to Nrf2 (Nrf2 KD) had decreased expression of GCLM and NQO1, two Nrf2‐mediated antioxidant genes (Figure 1A). GLCM, the regulatory subunit of Glutamate‐cysteine ligase, synthesizes glutathione (GSH), the most abundant cellular redox buffer. Total GSH was induced in control cells with CSE except at the highest dose (Figure 1B). In contrast, total GSH was decreased by >50%, a decline known to induce cell death, in Nrf2 KD cells exposed to CSE (Figure 1B, Han et al., 2003) with an associated magnified increase in ROS (Figure 1C), protein carbonylation (Figure 1D), a measure of oxidative damage, and a lowered threshold for cell death (Figure 1E). A similar, magnified increase of cell death from CSE was seen in Nrf2 KD iPS‐RPE cells compared to controls (Figure 1F).

### 2.2 **Nrf2** **KD does not alter mitochondrial antioxidant enzyme abundance**

**FIGURE 2:**
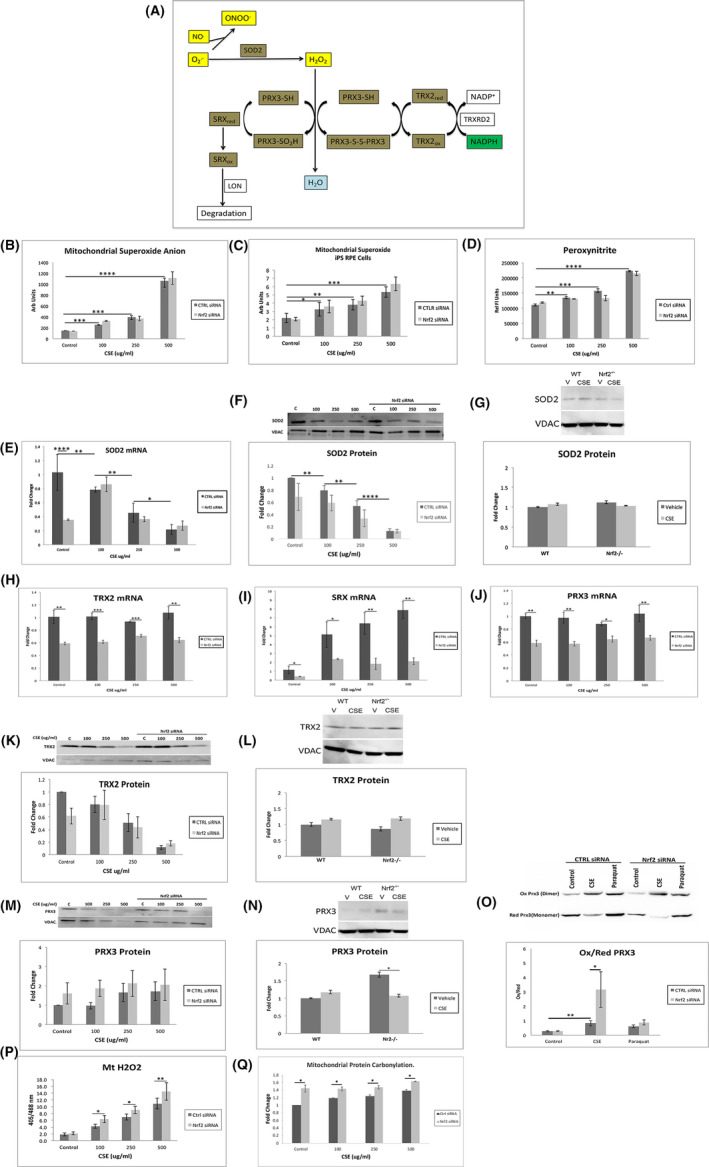
Mitochondrial antioxidant response with CSE and Nrf2 deficiency. A, Diagram of the TRX 2 system. Superoxide anion (O_2_^−^) reacts with nitric oxide (NO) to produce peroxynitrites (ONOO^−^). SOD2 reduces O_2_^−^ to H_2_O_2_. Homodimer PRX3 reduces H_2_O_2_ to H_2_O to produce a disulfide bond with the other PRX3 subunit (PRX3‐S‐S‐PRX3), which is reduced by TRX2 (TRX2_red_) to form oxidized (TRX2_ox_). When hyperoxidized PRX3 (PRX3‐SO_2_H) is formed after reacting with two H_2_O_2_ molecules, it can be reduced by SRX (SRX_red_), which translocates from the cytoplasm. Oxidized SRX (SRX_ox_) is degraded by Lon protease (LON). Nrf2 regulated genes are indicated by brown highlight. B, Mitochondrial O_2_^−^ using Mitosox assay, increases with CSE, but is not further increased with Nrf2 KD. C, A similar pattern of increased O_2_^−^ with CSE was not further increased with Nrf2 KD is seen in iPS‐RPE cells. D, Peroxynitrite levels increase with CSE without further increase with Nrf2 KD. E, SOD2 mRNA decreases with CSE. The expression, by RT‐qPCR, is decreased by Nrf2 KD only in unstimulated cells. F, Representative western blot and graph of decreased SOD2 protein from mitochondrial extracts with CSE without further decrease by Nrf2 KD. VDAC was used for normalization. G, Representative western blot of SOD2 protein from mitochondrial extracts in RPE cells from wild type (WT) or Nrf2^−/−^ mice given either Intravitreal vehicle or CSE. Mitochondrial H_2_O_2_ increases with CSE and Nrf2 deficiency due to a limited response from the TRX2 system. Expression of H) TRX2, I) SRX, and J) PRX3 mRNA by RT‐qPCR. K, Representative western blot of TRX2 protein from mitochondrial extracts with decrease after CSE, but no further decline with Nrf2 KD. L, Representative western blot of TRX2 protein from mitochondrial extracts in RPE cells from wild type (WT) or Nrf2^−/−^ mice given either intravitreal (IVT) vehicle or CSE. M, Representative western blot of PRX3 protein from mitochondrial extracts showing no change with CSE, with or without Nrf2 KD. N, Representative western blot of PRX3 protein from mitochondrial extracts in RPE cells from wild type (WT) or Nrf2^−/−^ mice given either IVT vehicle or CSE. PRX3 protein is induced in WT mice given IVT CSE, but decreases to levels seen in either WT or Nrf2^−/−^ mice given either IVT vehicle or CSE. O, Representative western blot of oxidized to reduced PRX3 in ARPE‐19 cells given CSE 250 μg/ml relative to vehicle. Paraquot serves as a positive control. P, Mitochondrial H_2_O_2_, measured by relative fluorescence after transfecting with roGFP2‐Orp1 plasmid, is increased by CSE, an effect that is magnified by Nrf2 KD. *p < 0.05, **p < 0.01, ***p < 0.001

Cigarette smoke can injure mitochondria including in the RPE. Mitochondria produce 90% of cellular ROS, initially as O_2_^−^ (Grivennikova et al., 2010, Figure 2A). The increased ROS by CSE measured by DHE assay, which detects O_2_^−^ among other ROS, suggests that mitochondria are the likely source. Indeed, CSE induced mitochondrial O_2_^−^ using Mitosox, compared to vehicle‐treated cells (Figure 2B). However, mitochondrial O_2_^−^ was not further increased with Nrf2 KD relative to controls in both ARPE‐19 and iPS‐RPE cells after CSE (Figure 2C). O_2_^−^ can react rapidly with nitric oxide to form peroxynitrites (ONOO^−^), which damage mitochondria (Szabo et al., 2007). Like O_2_^−^, ONOO^−^ was increased at similar levels in controls and Nrf2 KD cells with CSE (Figure 2D).

The relative increase in O_2_^−^ with CSE suggests that mitochondrial superoxide dismutase 2 (SOD2), which converts O_2_^−^ to H_2_O_2_ and can be Nrf2 regulated (Thimmulappa et al., 2002), is insufficiently induced to neutralize O_2_^−^. While SOD2 mRNA was decreased with Nrf2 KD relative to controls, SOD2 mRNA levels were decreased to a similar degree in Nrf2 KD and control cells with CSE (Figure 2E). SOD2 protein in mitochondrial extracts was decreased by CSE at similar levels in controls and Nrf2 KD cells (Figure 2F), and in mitochondrial extracts from the RPE of Nrf2^−/−^ mice and controls after intravitreal (IVT) injection of CSE 125 μg/ml (Figure 2G).

Mitochondrial H_2_O_2_ is neutralized principally by the Thioredoxin 2 (TRX2) system (Stowe & Camara, 2009). Peroxiredoxin 3 (PRX3), a homodimer, reduces H_2_O_2_ to water, and oxidized PRX3 is reduced by TRX2 (Figure 2A), (Biteau et al., 2003; Hall et al., 2011; Jeong et al., 2012). Occasionally, PRX3 gets inactivated after being hyperoxidized unless it is reduced by sulfiredoxin (SRX) (Noh et al., 2009). The mRNA of TRX2, SRX, and PRX3 was decreased by Nrf2 KD relative to controls treated with CSE, suggesting that they can be Nrf2 regulated, as reported (Bae et al., 2009; Dimauro et al., 2012; Harris & Hansen, 2012; Lim et al., 2016), (Figure 2H‐J). However, TRX2 protein in mitochondrial extracts was decreased to a similar degree in controls and Nrf2 KD cells after CSE (Figure 2K), and in RPE mitochondrial extracts from Nrf2^−/−^ and control mice given IVT CSE (Figure 2L). While abundant in the cytoplasm, SRX protein was not detected in mitochondrial extracts of either control or Nrf2 KD cells or in the RPE of WT or Nrf2^−/−^ mice given IVT CSE (data not shown).

PRX3 protein in mitochondrial extracts was unchanged in control and Nrf2 KD cells by CSE relative to vehicle (Figure 2M) and in mitochondrial extracts from the RPE of WT mice given IVT CSE. In Nrf2^−/−^ mice, PRX3 was elevated in vehicle‐injected mice relative to WT controls, and was decreased after CSE to levels of WT mice (Figure 2N). Importantly, oxidized PRX3 was increased after CSE in controls, and its level was magnified by Nrf2 KD (Figure 2O). After transfecting cells with the roGFP2‐Orp1 plasmid, mitochondrial H_2_O_2_ was increased after CSE, and further increased with Nrf2 KD (Figure 2P), and increased protein carbonylation, an oxidation marker, of mitochondrial proteins (Figure 2Q), correlating with increased oxidized PRX3.

### 2.3 **Nrf2** **KD decreases the isocitrate dehydrogenase (IDH) shuttle, the pentose phosphate pathway (PPP), and NADPH**

NADPH supplies reducing equivalents for the TRX2 system. Since NADPH is unable to enter the mitochondria from the cytoplasm, mitochondria derive NAPDH predominantly from NADH by nicotinamide nucleotide transhydrogenase (NNT), and the rest by Malic enzyme 3 (ME3) and glutamate dehydrogenase (GLUD1) (Nickel et al., 2015; Ronchi et al., 2016). NNT mRNA was not influenced by CSE, but was decreased with Nrf2 KD relative to controls at higher CSE doses (Figure S1A). However, NNT protein was unchanged by CSE or Nrf2 KD, except for a marked, equal decline at the highest CSE dose (Figure S1A). Both ME3 and GLUD1 mRNA and protein levels were unchanged in controls and Nrf2 KD cells treated with CSE (Figure S1B,C).

**FIGURE 3:**
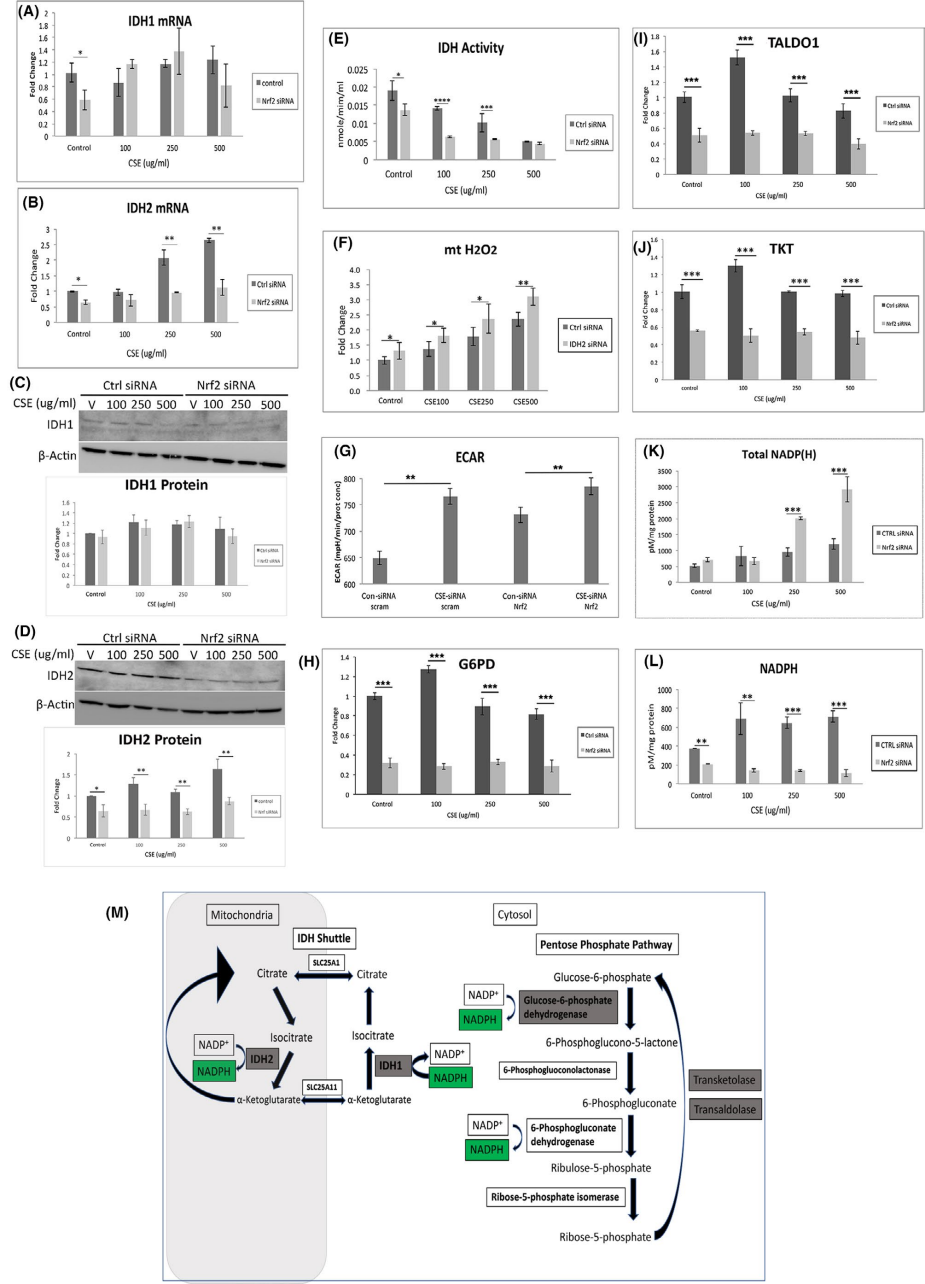
IDH shuttle and PPP are decreased with Nrf2 deficiency. Expression of A) IDH1 and B) IDH2 mRNA using RT‐qPCR. C, Representative western blot and graph of IDH1 showing no change with either CSE or Nrf2 KD. D, Representative western blot and graph of IDH2 showing decreased abundance with Nrf2 KD. E, IDH activity is decreased by CSE and is exaggerated with Nrf2 KD. F, Mitochondrial H_2_O_2_, as measured by relative fluorescence after transfecting with roGFP2‐Orp1 plasmid, is increased by CSE, an effect that is magnified by IDH2 KD. G, Aerobic glycolysis is increased with Nrf2 KD. Graph of increase in ECAR with CSE or Nrf2 KD relative to controls treated with vehicle. ECAR is not magnified with Nrf2 KD. The expression of H) G6PD, I) TALDO1, and J) TKT is decreased by Nrf2 KD using RT‐qPCR. K, Total NADP(H), comprised of NADP +NADPH, is not induced by CSE, but is increased by Nrf2 KD at the higher CSE doses. L, NADPH levels are reduced by Nrf2 KD relative to controls given CSE. *p < 0.05; **p < 0.01; ***p < 0.001; ****p < 0.00001. M, Diagram of the indirect NADPH transfer from the cytosol to mitochondria by the IDH shuttle. NADPH is generated by the PPP, as a result of reactions from Glucose‐6‐phosphate dehydrogenase and 6‐Phosphogluconate dehydrogenase. Transketolase and Transaldolase can convert ribose‐5‐phosphate to glucose‐6‐phosphate, which fuels the PPP. IDH1 converts α‐ketoglutarate to isocitrate and is converted to citrate, which is transported by SLC25A1 to the mitochondria. Mitochondrial IDH2 uses isocitrate to generate α‐ketoglutarate and NADPH. With Nrf2 KD, Nrf2 regulated genes (green highlight) are decreased, which decreases the PPP activity and NADPH generation, and decreases IDH activity to decrease mitochondrial NADPH

The IDH shuttle can indirectly provide NADPH from the cytoplasm to mitochondria (Jiang et al., 2016). IDH1 generates isocitrate/citrate by oxidizing cytosolic NADPH, and citrate enters the mitochondria by SLC25A1. Mitochondrial IDH2 uses isocitrate/citrate to generate α‐ketoglutarate and NADPH. IDH1 and IDH2 can be regulated by Nrf2 (Bai et al., 2018; Liu et al., 2020). While IDH1 mRNA was decreased in Nrf2 KD cells compared to controls without CSE, IDH1 mRNA and protein were not influenced by CSE in controls or Nrf2 KD cells (Figure 3A,C). In contrast, IDH2 mRNA and protein were decreased by Nrf2 KD with and without CSE (Figure 3B,D). Importantly, IDH activity was decreased by CSE, an effect that was magnified by Nrf2 KD (Figure 3E). Citrate abundance or transport, which could influence the IDH shuttle, were not altered. The citrate transporter SLC25A1 was not influenced in the mitochondrial fraction by Nrf2 KD with or without CSE (Figure S2A). Likewise, aconitase activity in the mitochondrial fractions, a source of citrate, while slightly induced by CSE, was not decreased by Nrf2 KD (Figure S2B). Finally, mitochondrial H_2_O_2_ was increased by CSE and magnified by IDH2 KD (Figure 3F).

Cytoplasmic NADPH is predominantly generated by the PPP. With mitochondrial impairment, the RPE converts to aerobic glycolysis, which can augment both ATP production and PPP activity to increase NADPH. Indeed, the extracellular acidification rate (ECAR) was increased by CSE in control and Nrf2 KD cells to a similar degree (Figure 3G). While Nrf2 can regulate the expression of genes involved in glycolysis, we found that 6‐phosphofructo‐2‐kinase/fructose‐2,6‐biphosphatase 3, hexokinase‐1 and −2, and pyruvate dehydrogenase 1 were not altered by CSE or Nrf2 KD (data not shown) (Kuosmanen et al., 2018; Ohl et al., 2018). Likewise, since Hypoxia inducible factor‐1a (HIF1α0 can regulate glycolysis, we next found that while HIF1α was induced by CSE only at the higher doses, it was not further altered by Nrf2 KD (Figure S3). However, the expression of PPP genes Glucose‐6‐phosphate dehydrogenase, transaldolase (TALDO1), and Transketolase, which all can be Nrf2 regulated (Chang et al., 2018; Lee et al., 2003; Ohl et al., 2018; Xu et al., 2016), were decreased with Nrf2 KD with or without CSE (Figure 3H‐J). While total NADP(H), comprised of NADP plus NADPH, increased with CSE, NADPH was decreased with Nrf2 KD (Figure 3K,L).

**FIGURE 4:**
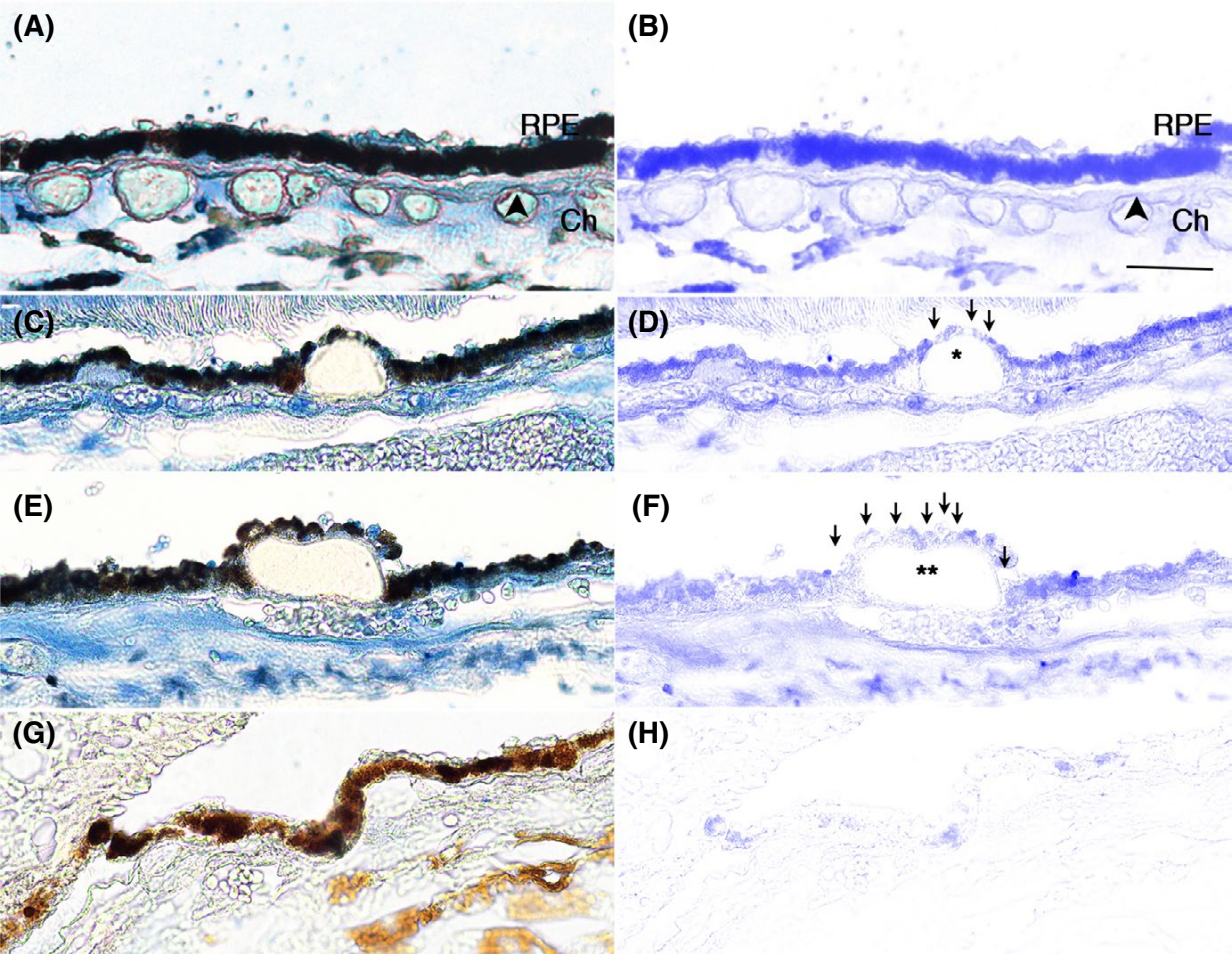
TALDO1 is decreased in macular RPE of a 61 year old male with dry AMD. A, Morphologically normal RPE with strong, consistent blue immunolabeling for TALDO1. B, TALDO1 immunolabeling after Nuance software has subtracted melanin. C, TALDO1 immunolabeling is slightly decreased in dysmorphic RPE overlying intermediate druse (*) relative to normal appearing adjacent cells. D, TALDO1 immunolabeling is better visualized after melanin has been subtracted. Arrows point to RPE with reduced labeling. E, Dysmorphic RPE overlying large druse (**) have reduced TALDO1 immunolabeling relative to adjacent normal RPE. F, TALDO1 immunolabeling is highlighted after melanin has been removed. Arrows point to RPE with reduced labeling. G) and H) IgG control without immunolabeling with and without Nuance subtraction. Bar = 25 μm. RPE, retinal pigment epithelium; Ch, choroid. Arrowhead, Bruch's membrane

To provide relevance to AMD, the distribution of TALDO1 was examined in the RPE of AMD maculas. In morphologically normal RPE of age‐matched controls and dry AMD maculas, TALDO1 immunolabeling was robust and consistent across the RPE (Figure 4A,B) including when overlying small drusen (Figure 4C,D). However, TALDO1 labeling in dysmorphic RPE, particularly overlying large drusen, was decreased (Figure 4E,F; Table 1).

**TABLE 1:** Donor information for TALDO1 immunohistochemistry

Donor	Age	Gender	Race	D‐E (h)	Macula			Periphery		
					BrM	Small Drusen	Large Drusen	BrM	Small Drusen	Large Drusen
Unaffected										
1	41	F	C	30	Yes	NP	NP	Yes	NP	NP
2	30	M	B	32	Yes	NP	NP	Yes	NP	NP
3	54	M	C	13	Yes	NP	NP	Yes	NP	NP
4	87	M	C	24	Yes	NP	NP	Yes	NP	NP
5	83	F	C	13	Yes	NP	NP	Yes	NP	NP
Early AMD										
1	77	F	C	93	Yes	Yes	No	Yes	NP	NP
2	61	M	C	20	Yes	Yes	Yes	Yes	NP	NP
3	96	M	C	05	Yes	Yes	No	Yes	NP	NP
4	65	F	C	04	Yes	Yes	No	Yes	NP	NP
5	62	F	C	29	Yes	NP	Yes	Yes	NP	NP
6	94	F	C	08	Yes	NP	Yes	Yes	NP	NP

Note

TALDO1 immunostaining in the RPE overlying thickened Bruch's membrane (BrM), small, or large drusen. B, Black; C, Caucasian; D‐E, death to enucleation; F, Female; M, Male; NP, not present.

### 2.4 **Nrf2** **KD impairs mitochondrial function**

**FIGURE 5:**
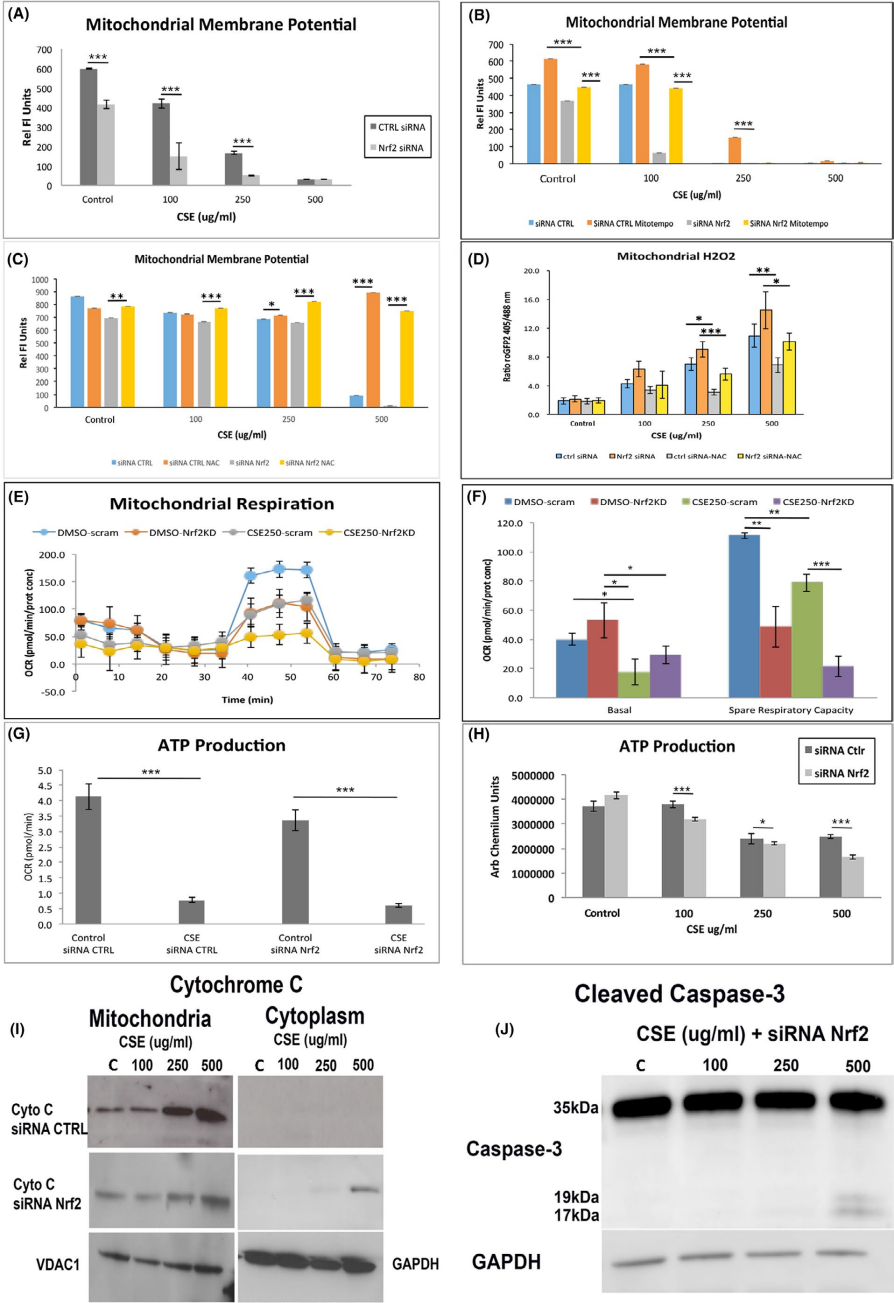
Mitochondrial dysfunction is magnified with Nrf2 KD. A, Mitochondrial membrane potential (MMP), as measured by TMRM, is decreased by CSE, and magnified with Nrf2 KD. B, Mitotempo, which scavenges O_2_^−^, rescues MMP at low CSE doses with Nrf2 KD, but has no effect at high doses. C, NAC, which neutralizes H_2_O_2_, rescues MMP. D, Mitochondrial H_2_O_2_, as measured by relative fluorescence after transfecting with roGFP2‐Orp1 plasmid, is increased by CSE, an effect that is magnified by Nrf2 KD, and is neutralized by NAC, including high CSE doses. E, Tracing of oxygen consumption rate (OSR) using Seahorse in Nrf2 KD and control ARPE‐19 cells, with or without CSE 250 μg/ml. F, Graph of decreased basal OSR after CSE in Nrf2 KD cells relative to controls given vehicle. The spare respiratory capacity is reduced by CSE and is magnified by Nrf2 KD. G, ATP production, by Seahorse assay, is decreased by either CSE or Nrf2 KD. H, ATP production, measured by Cell Titer Glo, is decreased in control cells by CSE, an effect that was magnified with Nrf2 KD. I, Representative western blot of cytochrome C in mitochondrial and cytoplasmic extracts after CSE in control and Nrf2 KD cells. Cytochrome C release to the cytoplasm is seen at the higher CSE doses with Nrf2 KD. VDAC1 and GAPDH were used to normalize mitochondrial and cytoplasmic expression, respectively. J, Representative western blot of cleaved caspase 3 in Nrf2 KD cells given CSE. The cleaved form is seen at the higher CSE doses. GAPDH is used to normalize expression. *p < 0.05; **p < 0.01; ***p < 0.001

With increased mitochondrial ROS, the mitochondria are susceptible to injury (Grivennikova et al., 2010). Indeed, MMP was decreased by CSE in controls, an effect that was magnified by Nrf2 KD (Figure 5A). Treatment with Mitotempo, a mitochondria‐specific SOD mimetic, restored MMP in controls and Nrf2 KD cells at low, but not high CSE doses (Figure 5B). Treatment with N‐acetyl cysteine (NAC) neutralized both mitochondrial and cytoplasmic H_2_O_2_, and restored MMP in Nrf2 KD cells even at the highest CSE dose (Figure 5C), which was commensurate with decreased mitochondrial H_2_O_2_ (Figure 5D).

Oxygen consumption rates (OCR) were also reduced by CSE and Nrf2 KD (Figure 5E). While basal OCR was unaltered, the reserve capacity was decreased by CSE, and further decreased with Nrf2 KD (Figure 5F). Likewise, ATP was decreased by CSE in controls, and was worsened by Nrf2 KD (Figure 5G,H) which correlated with decreased electron transport chain (ETC) complex I‐IV activities by CSE in controls (Figure S4A‐D) and in the RPE of WT mice given IVT CSE (Figure S4E‐H). Compared to controls, ETC I and II activities were further decreased in Nrf2 KD cells by CSE, and in the RPE of Nrf2^−/−^ given IVT CSE.

### 2.5 **Nrf2** **KD cells exposed to CSE die by intrinsic apoptosis**

Severe mitochondrial dysfunction can initiate the intrinsic apoptotic pathway, inducing the release of cytochrome c, a key initiating apoptotic event to the cytosol (Gottlieb et al., 2003). CSE induced cytochrome c in the mitochondria, but not the cytoplasm (Figure 5I). Cytoplasmic cytochrome c became barely detectable in Nrf2 KD cells after CSE 250μg/ml, the threshold of increased cell death, and was obvious with CSE 500 μg/ml. Furthermore, cleaved caspase 3, indicative of the committed apoptotic stage, was first detected in Nrf2 KD cells treated with CSE 250 μg/ml, and was increased with CSE 500 μg/ml (Figure 5J). Cleaved caspase 3 was not seen in control ceels treated with CSE (not shown).

## 3 DISCUSSION

Excessive ROS can cause cell injury and death. Oxidative stress from cigarette smoke directly damages mitochondria, a phenomenon that we observed in RPE cells (Anbarasi et al., 2005; Smith et al., 1993). Nrf2, which coordinates a comprehensive antioxidant response, (Sykiotis & Bohmann, 2010) decreases in several oxidative stress‐related diseases including AMD (Suh et al., 2004; Suzuki et al., 2008; Wang et al., 2014). Since the subcellular location of Nrf2’s antioxidant activity is understudied, and since mitochondria produce the majority of cellular ROS, we investigated the impact of decreased Nrf2 on the mitochondrial antioxidant response. We studied RPE cells because they have robust mitochondrial and antioxidant activity, and their mitochondria are injured and Nrf2 is decreased in AMD. Since no ROS assay is free of artifact (Kowaltowski, 2019), we used multiple approaches to measure several ROS species and key elements of the antioxidant response. Herein, we find that Nrf2 did not influence mitochondrial antioxidant enzyme abundance, but instead, regulated NADPH through the expression of genes in the PPP, the principal source of cytoplasmic NADPH, and the IDH shuttle, which together indirectly provide NADPH to the mitochondria for the TRX2 system to neutralize H_2_O_2_ (Jiang et al., 2016). With Nrf2 deficiency, mitochondria were damaged by inadequately neutralized H_2_O_2_ after CSE stress, which lowered the threshold for cell death by activating intrinsic apoptosis.

The TRX2 system including TRX2, SRX, and PRX3, which can be Nrf2 regulated, (Arner, 2009; Niso‐Santano et al., 2010) principally neutralizes mitochondrial H_2_O_2_ (Han et al., 2003; Stowe & Camara, 2009). Their abundance was unchanged by Nrf2 KD. Rather, NADPH, which provides reducing equivalents for the TRX2 system to prevent reduction of PRX3, was decreased, resulting in increased oxidized PRX3. In mitochondria, approximately 45% of NADPH is generated by NNT, with significant contributions from ME3 and GLUD1 (Ronchi et al., 2016). In our experiments, they were also unaltered by Nrf2 KD. With low NADH, NNT activity can reverse itself under severe metabolic stress when NADH is needed as substrate for ETC complex I by generating NADH from NADPH. Since this reversal requires abundant NADPH, (Nickel et al., 2015) this scenario is unlikely because NADPH was decreased with Nrf2 KD. Interestingly, because the WT and Nrf2^−/−^ mice are in a C57BL6J background, they carry an NNT mutation (Ronchi et al., 2013). Despite impaired NNT, Nrf2 deficiency did not impair the abundance of TRX2 elements with the exception of higher baseline PRX3 levels in Nrf2^−/−^ mice relative to WT mice. Future studies using mice with the C57BL6 background must consider the effect of NNT on oxidative stress.

NADPH does not cross into mitochondria. However, cytoplasmic NADPH can indirectly provide mitochondrial NADPH through the IDH1‐IDH2 shuttle after isocitrate/citrate produced by cytosolic IDH1 enters the mitochondria by SLC25A1 (Heisterkamp et al., 1995, Figure 3M). IDH2 then uses isocitrate/citrate to generate α‐ketoglutarate and NADPH in the mitochondria (Jiang et al., 2016). Low cytoplasmic NADPH slows the shuttle by reducing the conversion of α‐ketoglutarate to isocitrate by cytoplasmic IDH1. In addition, low mitochondrial IDH2 reduces mitochondrial NADPH production. Despite containing an ARE site in its promoter (Bai et al., 2018), IDH1 was not influenced by Nrf2 KD. However, IDH2, which is regulated by Nrf2 through SIRT3 (Liu et al., 2020), was decreased by Nrf2 deficiency. Importantly, IDH shuttle activity was decreased by both decreased IDH2 and NADPH from the reduced expression of Nrf2‐mediated genes in the PPP, the main producer of cytoplasmic NADPH (Chang et al., 2018; Lee et al., 2003; Ohl et al., 2018; Xu et al., 2016). Glucose‐6‐phosphate dehydrogenase, which converts glucose‐6‐phosphate to 6‐phosphoglucono‐5‐lactone, reduces NADP+to NADPH. The non‐oxidative PPP genes Transketolase and TALDO1, which convert ribose‐5‐phosphate to glucose‐6‐phosphate, are relevant for NADPH production by the PPP. For example, Transaldolase mutations reduce NADPH production by the PPP (Perl et al., 2006). Ultimately, the NADPH supply was insufficient to reduce PRX3 by TRX2 and SRX, which impaired H_2_O_2_ neutralization by PRX3. While inadequately neutralized O_2‐_ and ONOO^−^ contributed to mitochondrial impairment, as others have found, (Radi et al., 1994; Szabo et al., 2007) the markedly elevated H_2_O_2_ can explain the exaggerated mitochondrial injury with Nrf2 KD after CSE. The rejuvenated MMP with NAC supports the prior observation that H_2_O_2_ can directly injure mitochondria (Colom et al., 2007).

Our findings are relevant to AMD. We found that TALDO1, an Nrf2 regulated gene (Lee et al., 2003), had reduced immunostaining in dysmorphic RPE overlying drusen, a hallmark lesion in dry AMD, in the macula of AMD globes, a pattern we observed with Nrf2 (Wang et al., 2014). In early AMD, mitochondrial injury is a well‐established pathogenic factor that develops in the RPE before the retina, and prior to overt vision loss (Karunadharma et al., 2010; Lin et al., 2011; Nordgaard et al., 2006, 2008; Terluk et al., 2015). Our group found with RNA‐seq of the RPE and retina from AMD globes, that genes involved in mitochondrial function in the RPE were among the most highly differentially expressed genes relative to other processes implicated in early AMD (Wang et al., 2018). This finding highlights its importance relative to other processes that contribute to AMD pathobiology. Thus, the impaired mitochondrial antioxidant response by Nrf2 deficiency could activate the intrinsic apoptotic pathway, and lower the threshold for cell death. This finding may be relevant to AMD because apoptosis is one mechanism of RPE death (Del Priore et al., 2002; Dunaief et al., 2002).

With Nrf2 deficiency, decreased NADPH from an impaired IDH shuttle and decreased expression of genes in the PPP increased mitochondrial H_2_O_2_ beyond the threshold of effective neutralization by the TRX2 system, contributed to mitochondrial injury with CSE. Ultimately, the mitochondrial injury was severe enough to induce apoptotic cell death. Nrf2 directly binds to mitochondria, although this role is unclear (Strom et al., 2016). Given that Nrf2 is decreased in the RPE with AMD, further characterization of Nrf2’s role on the mitochondrial antioxidant response and a mechanistic link to AMD is warranted.

## 4 EXPERIMENTAL PROCEDURES

### 4.1 Cell culture

The maintenance of human ARPE‐19 cells has been described (Dunn et al., 1996). Human induced Pluripotent stem cells (CB6.2 line) were maintained in TeSR1 (STEMCELL Technologies) for 10 days, and placed in differentiation medium for 50 days, and plated onto Matrigel‐coated plates in RPE medium as described (Maruotti et al., 2013). RPE cells were grown for 3 months and exposed to Cigarette Smoke Extract (CSE; Murty Pharmaceuticals) for 24 hr.

### 4.2 Measurement of ROS

Cellular ROS, which measures predominantly superoxide anion (O_2_^−^), but also other ROS, was measured after labeling cells with 10 µM dihydroethidium (DHE; Thermo Fisher Scientific), and measuring ethidium‐DNA fluorescence. Mitochondrial O_2_^−^ and peroxynitrite were measured with the Mitosox Red assay (Thermo Fisher) and the peroxinitrite assay (Abcam), respectively. Mitochondrial H_2_O_2_ was quantified using mito‐roGFP2‐Orp1 fusion protein as after transfecting cells with a plasmid encoding roGFP2‐Orp1, a redox sensitive biosensor targeted to the mitochondrial matrix (Roma et al., 2018) using Lipofectamine 2000. roGFP2 fluorescence was measured using a microplate reader (BMG LABTECH).

### 4.3 RNA extraction

Total RNA was extracted using the RNeasy Mini‐kit (Qiagen Inc.). RNA quality was confirmed with the Agilent Bioanalyzer (Agilent Technologies).

### 4.4 Quantitative real‐time RT‐PCR (RT‐qPCR)

Reverse transcription was performed using random hexamers and MultiScribe reverse transcriptase (Applied Biosystems). RT‐qPCR analyses were performed using Assay‐on‐Demand primers and probe sets (Applied Biosystems) with a StepOnePlus Taqman system.

### 4.5 Transfection of small interfering RNA (siRNA)

siRNA targeting human Nrf2 and control siRNA (Applied Biosystems) were used (Singh et al., 2006) after transfection with either 15 nM siRNA in Lipofectamine 2000 (Thermo Fisher) for 24 h.

### 4.6 Western analysis

Membranes were incubated with primary antibody (rabbit polyclonal anti‐SOD2 (1:500, Abcam); rabbit polyclonal TRX2 (1:500, Santa Cruz Biotechnologies); Goat Polyclonal anti‐SRX (1:500, Abcam); rabbit polyclonal anti‐PRX‐3 (1:500, Abcam), rabbit polyclonal anti‐NNT (1:10000; Thermo Fisher), rabbit monoclonal anti‐ME3 (1:500; Abcam), rabbit monoclonal anti‐GLUD1 (1:500; Cell Signaling), rabbit polyclonal anti‐IDH1 (1:100; Cell Signaling), rabbit monoclonal anti‐IDH2 (1:500; Cell Signaling), rabbit polyclonal anti‐SLC25A (1:2000, Thermo Fisher), mouse monoclonal anti‐HIF1a (1:1000 BD Biosciences), mouse monoclonal anti‐cytochrome C (1:200; Biovision), rabbit monoclonal anti‐cleaved caspase 3 (1:500; Cell Signaling) and then the appropriate horseradish peroxidase conjugated secondary antibody (1:5000, Abcam). For oxidized/reduced PRX3 western blot, cells were treated with CSE (250 μg/ml), DTT (5 mM), paraquot (25 µM), or DMSO for 24 h, washed, and then treated with 100 ml of alkylation buffer containing 100 mM NEM at room temperature (Roede et al., 2018). Cell lysates were separated on 4–12% bis‐tris SDS‐PAGE gel and transferred to a nitrocellulose membrane. Membranes were incubated with anti‐PRX3 (1:500; Abcam) and secondary anti‐rabbit antibody conjugated with horseradish peroxidase (1:500; Abcam). Signals from western blots were detected with a chemiluminescence detection system (Supersignal West Pico Chemiluminescence, Thermo Fisher). Blots were imaged with an ImageQuant LAS4000 scanner (GE Healthcare, Inc). Band intensity is reported as arbitrary densitometric units.

### 4.7 Aconitase activity assay

The aconitase activity assay was performed with a kit from Biovision.

### 4.8 Celltiter‐Glo ATP assay

ATP content was determined using the CellTiter‐Glo (CTG) ATP assay (Promega, Inc.).

### 4.9 Cell viability

Cell viability was evaluated with the Propidium Iodide (PI) Viability Assay (Thermo Fisher).

### 4.10 Electron transport chain activity assay

Electron transfer chain (ETC) activity of complexes I‐IV were measured using activity assays (BioVision).

### 4.11 Glutathione assay

Glutathione assay was performed using the Glutathione Fluorometric Assay Kit (Biovision).

### 4.12 IDH Activity assay

IDH activity was measured using the IDH activity assay (Sigma‐Aldrich).

### 4.13 Lactate assay

Lactate was measured using a kit from BioVision.

### 4.14 Mitochondrial membrane potential (MMP) assay

MMP was measured using the TMRM kit (Thermo Fisher).

### 4.15 NADP/NADPH assay

NADP/NADPH was quantified using a kit from BioVision.

### 4.16 Protein carbonylation assay

Protein carbonylation was quantified using ELISA (Cell Biolabs, Inc).

### 4.17 Seahorse assay

Mitochondrial oxygen consumption rate (OCR) and extracellular acidification rate (ECAR) were assessed using a Seahorse Bioscience XFe96 analyzer (Agilent) in combination with the Seahorse Bioscience XF Cell Mito Stress Test assay kit. Cells (4 × 10^4^ cells/well) were seeded in a 96‐well Seahorse plate, treated 24 h later with siRNA, and then with or without CSE for 24 h. The initial 35 min reading established the baseline followed by treatment with 1 μmol/L oligomycin, 1 μmol/Lcarbonyl cyanide phospho‐(p)‐trifluoromethoxy phenylhydrazone (FCCP), and 0.5 μmol/L rotenone/antimycin. The OCR and ECAR were calculated by the Seahorse XFe96 Wave software.

### 4.18 Intravitreal CSE treatment of mice

All experiments were conducted according to the ARVO Statement for the Use of Animals in Ophthalmic and Vision Research, and the IRB at Johns Hopkins Medical School approved the research. Male and female Nrf2‐deficient mice (Nrf2^−/−^; C57Bl6J background) and littermate controls, RD8 negative, were given 250 ug/ml CSE (1 μl) by intravitreal injection in one eye and DMSO (1 μl) in the contralateral eye using a microinjection pump (Harvard Apparatus). After sacrifice, eyes were enucleated, and the RPE was prepared for western analysis or mitochondrial activity assays by extracting protein.

### 4.19 Human tissue processing

The protocol adhered to the tenets of the Declaration for Helsinki for research involving human tissue. Human autopsy eyes (*n* = 11) were obtained from the Wilmer Eye Institute Pathology Lab (Table 1). Donors were classified as “unaffected” (*n* = 5) if they had no AMD history or histopathologic evidence of drusen. Early AMD donors (*n* = 6) had an AMD history and macular drusen. Small drusen were defined as having a diameter of 63 μm and large drusen as 125 μm. Eyes were fixed in 4% formaldehyde, paraffin embedded, and sectioned at 4 μm thickness.

### 4.20 Immunohistochemistry

After deparaffinizing sections, antigens were retrieved using the Target Retrieval System (Dako, Inc.). Sections were blocked with horse serum and incubated with rabbit polyclonal anti‐TALDO1 (1:50; Abcam) or rabbit IgG control overnight at 4℃, with biotinylated anti‐rabbit IgG (Vectastain^®^ABC‐AP Kit; Vector Labs), and then with ABC‐AP (Vector Labs). Slides were incubated with levamizole added to blue substrate working solution. Sections were imaged with a light microscope equipped with the Cri‐Nuance system (Perkin Elmer Corp, Inc.) to subtract out melanin pigment.

### 4.21 Statistical analysis

The difference between groups was statistically compared by Student's *t*‐test or analysis of variance (one‐way ANOVA) for groups of more than two with post hoc testing using Tukey‐Kramer HSD test. All statistical analyses were performed using JMP version 6.0.3 software (SAS). Experiments were conducted in triplicate and performed at least 3 times.

## CONFLICT OF INTEREST

MC: grant funding from Bayer Pharmaceuticals for unrelated project; JTH: grant funding and royalties from Bayer Pharmaceuticals for unrelated project; grant funding from Clover Pharmaceuticals for unrelated project; SD, MF‐B, MS, DS: None.

## AUTHOR CONTRIBUTIONS

MC (designed, performed, analyzed experiments, critiqued and approved of manuscript, agree to accuracy of the work), SD (designed, performed, analyzed experiments), LW (performed and analyzed experiments), TL (performed and analyzed experiments), MF‐B (performed and analyzed experiments), MS (performed and analyzed experiments), DS (interpreted experiments, wrote manuscript), JTH (analyzed and interpreted experiments, conceived of project topic, wrote manuscript). All critiqued, and approved of manuscript and agreed to the work's accuracy.

## Supporting information

Fig S1

Fig S2

Fig S3

Fig S4

## Data Availability

The data that support the findings of this study are available from the corresponding author upon reasonable request.
