# Supplementary figures and images for: Nrf2 deficiency decreases NADPH from impaired IDH shuttle and pentose phosphate pathway in retinal pigmented epithelial cells to magnify oxidative stress‐induced mitochondrial dysfunction
Source: Aging Cell. 2021 Jul 27;20(8):e13444. doi: 10.1111/acel.13444 (PMC8373343; doi:10.1111/acel.13444)

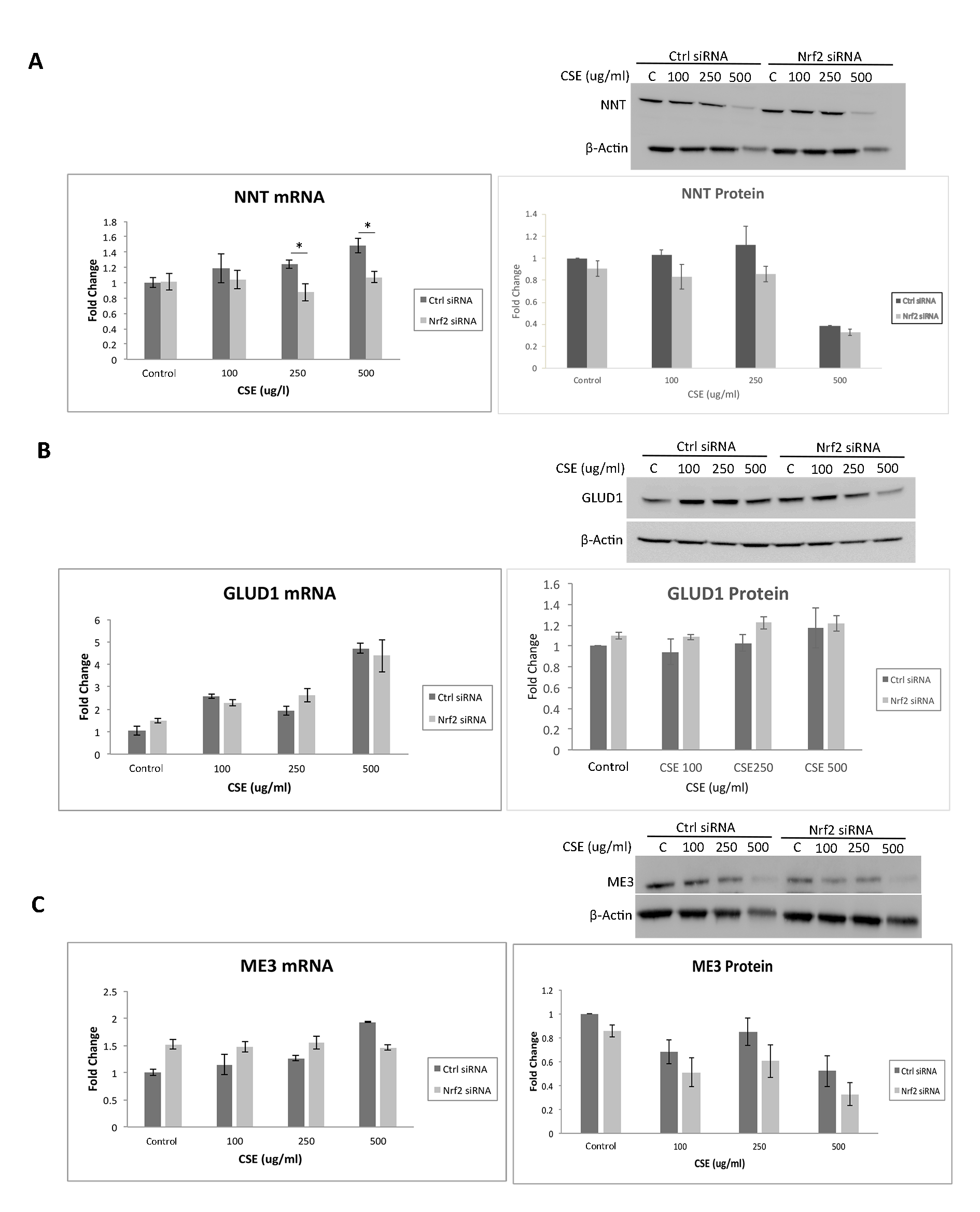

Supplement: Supplementary file 1 — Fig S1 [file ACEL-20-e13444-s001.tif]

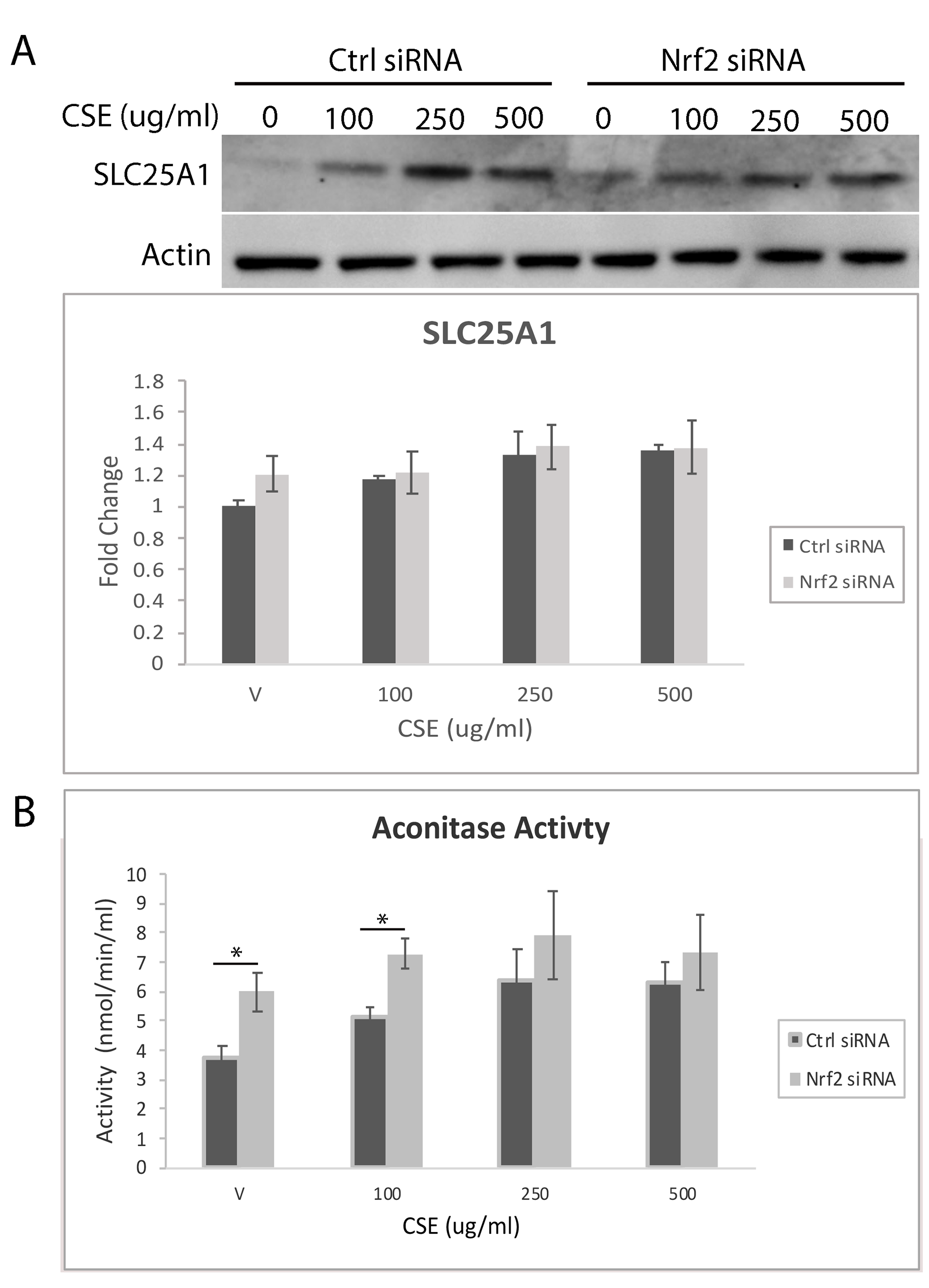

Supplement: Supplementary file 2 — Fig S2 [file ACEL-20-e13444-s003.tif]

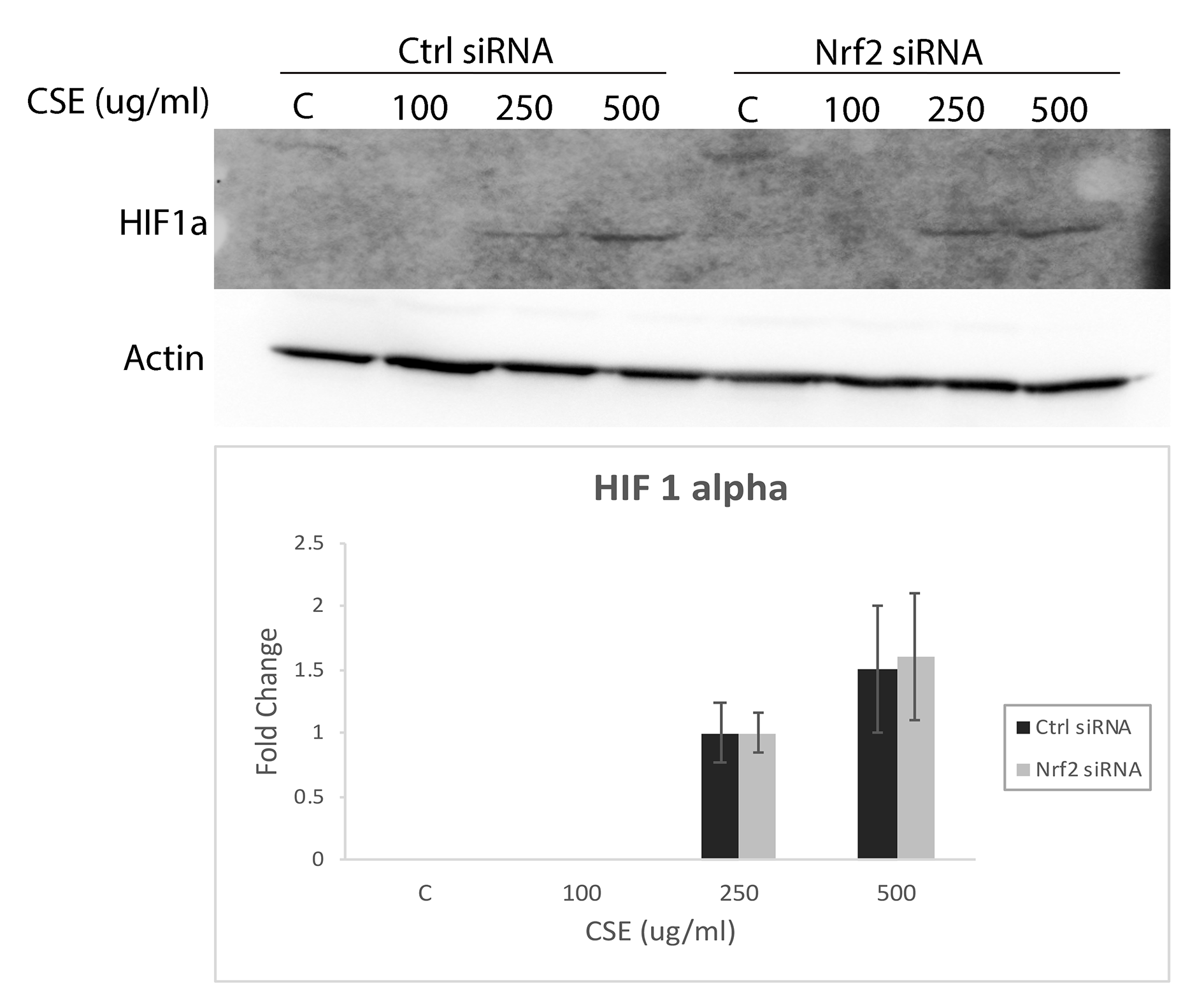

Supplement: Supplementary file 3 — Fig S3 [file ACEL-20-e13444-s002.tif]

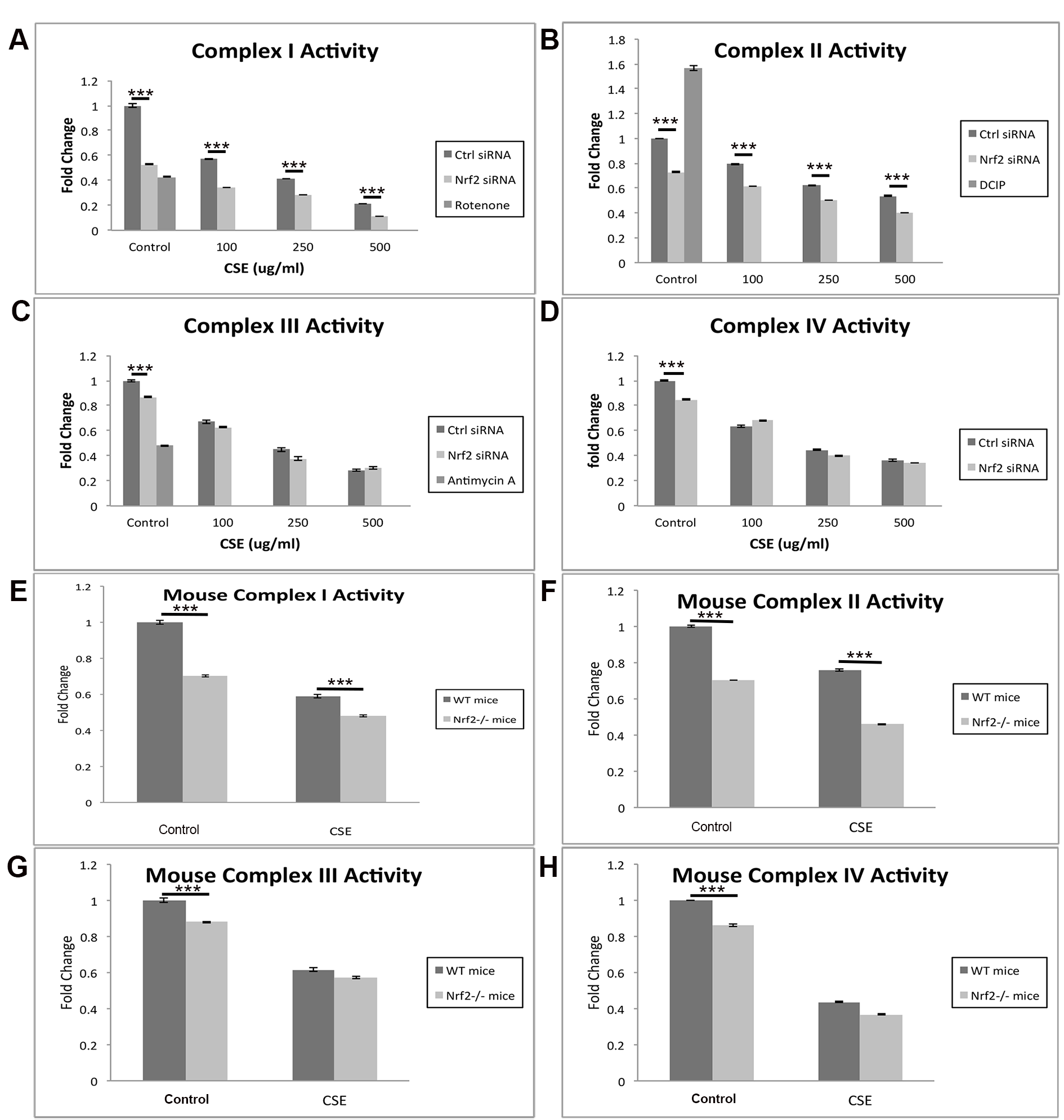

Supplement: Supplementary file 4 — Fig S4 [file ACEL-20-e13444-s004.tif]
